# Stinging Nettles as Potential Food Additive: Effect of Drying Processes on Quality Characteristics of Leaf Powders

**DOI:** 10.3390/foods10061152

**Published:** 2021-05-21

**Authors:** Swathi Sirisha Nallan Chakravartula, Roberto Moscetti, Barbara Farinon, Vittorio Vinciguerra, Nicolò Merendino, Giacomo Bedini, Lilia Neri, Paola Pittia, Riccardo Massantini

**Affiliations:** 1 Department for Innovation in Biological, Agro-Food and Forest Systems, University of Tuscia, 01100 Viterbo, Italy; swathi.nallan@unitus.it (S.S.N.C.); barbara.farinon@gmail.com (B.F.); vincigue@unitus.it (V.V.); merendin@unitus.it (N.M.); gbedini@unitus.it (G.B.); 2 Faculty of Bioscience and Technologies for Food, Agriculture, and Environment, University of Teramo, 01100 Viterbo, Italy; lneri@unite.it (L.N.); ppittia@unite.it (P.P.)

**Keywords:** *Urtica dioica* L., drying, powder, technical properties, functional properties

## Abstract

Stinging nettle (*Urtica dioica* L.) is a ubiquitous, multi-utility, and under-utilized crop with potential health benefits owing to its nutritional and bioactive components. The objective of the work is to produce powders by drying wild stinging nettle leaves as a storable, low-cost functional additive to be used in bakery and ready-to-cook products. Convective drying (CD) and freeze-drying (FD) were applied on unblanched (U) or blanched (B) leaves, which were then milled to nettle powders (NPs). The obtained NPs were evaluated for selected physicochemical (moisture, color), techno-functional (flow indices, hygroscopicity), and phytochemical (pigments, phenols) characteristics as well as mineral contents. Blanching improved mass transfer and reduced the oxidative degradation of pigments during drying, but it caused a loss of total phenols content, antioxidant activity, and potassium content. As for the drying method, CD resulted in better flow properties (i.e., Carr Index and Hausner Ratio), while FD retained better the color, pigments, magnesium content, phenolic, and antioxidant parameters. Overall, the evaluated processing methods resulted in different technological properties that can allow for better evaluation of NPs as a food additive or ingredient. Among the NPs, blanched and freeze-dried powders despite showing inferior technological properties can be recommended as more suitable ingredients targeted f or food enrichment owing to better retention of bio-active components.

## 1. Introduction

The rising consumer interest in functional foods and demand for ‘clean label’ food is pushing the food industry to rediscover the use of wild plants and potherbs. Their bioactive constituents and various physiological benefits at the molecular level has propelled research not only in the direction of medicine but also technologically to have industrially adaptable raw and auxiliary materials.

Among the vast selection of wild plants, *Urtica dioica* L., commonly known as stinging nettle of family Urticaceae, is a spontaneous, ubiquitous, and perennial plant and a known weed in intensive farming. It is traditionally used as medicine and is a multi-purpose commercial crop used for pharmaceutical extracts, textile fibers, and as colorant (chlorophyll, E140) [1,2,3]. Moreover, nettle leaves and extracts were found to contain various phytochemicals such as organic acids and phenolic compounds (e.g., flavonoids) that render them with diuretic, anti-diabetic, and anti-inflammatory activities [4,5]. However, water extract showed very little anti-hyperglycemic and antiglycation activities in induced-diabetic rats [6]. In fact, the major contributing constituents of nettle were found to be cyclic hydrophilic proteins [6], which are usually absent or in very low quantity in water extract. In addition, nettle leaves are good sources of lutein, ß-carotene, vitamins (A, C), minerals (Ca, K, Fe), and phenols in general [7,8,9], especially the well-documented chlorogenic acid, caffeic acid, and kaempferol-3-rutinoside [2], which can inhibit the glycation process [10]. These attributes make nettle leaves a potential source of bio-active components for utilization as a potherb and especially as whole ingredient in functional foods [11,12] as leaves, stems, and powders thereof.

Despite the various advantages mentioned, nettles remain under-utilized in the food sector due to seasonality and other reasons such as market stigma i.e., market resistance due to a consumer perception of nettle as famine/poor man’s food with low sensorial quality and undesirability due to stinging, as duly pointed by Shonte and de Kock (2017) [13]. As most vegetables and fruits, nettles are also perishable, requiring post-harvest operations to extend the storability and consumption period. Among various preservation technologies, an effective, viable, and widely used industrial process for seasonal foods to ascertain their economic value is drying. Drying with hot air or convection is a widely used method, even though it is energy intensive and detrimental to the product’s nutritional quality [14,15]. As for other available methods, freeze drying, although expensive, has been gaining commendable interest among food producers as an efficient technology that retains better nutritive and functional quality owing to the sublimation process at low temperatures [15,16].

With respect to the drying processes of nettle leaves, the available literature focused on drying and its optimization based on chemical and nutritional characteristics. Of these, Adhikari et al. (2016) [7] have comparatively characterized the proximate composition and selected functional components (tannins, polyphenols, and antioxidants) for solar dried leaves to that of wheat and barley flours as a potential ingredient. Shonte et al. (2020) [5] and Shonte and de Kock (2017) [13] in different studies observed that oven drying in general adversely affected the nutritional, functional, and sensorial properties of dried nettle leaves and their infusions, respectively. Similarly, Alibas (2007) [17] reported longer drying times in convection and microwave drying negatively affected the color of the dried nettle leaves. Movagharnejad et al. (2019) [18] found that at higher microwave power, shorter infrared lamp distance nettle leaves had shorter drying cycles and better release of phenolic compounds. Among different drying methods such as convection, freeze, microwave, air, and solar drying, Branisa et al. (2017) [16] recommended freeze drying as a preferable method for the optimal retention of pigments, antioxidant content, and phenolic content in nettle leaf powders. These results from different studies highlight that the drying processes, particularly those with high temperatures, may deteriorate the nutritional and functional quality of nettle products. A well-known pretreatment used to reduce the degradation of quality during drying is blanching, which as a stand-alone treatment was observed by Rutto et al. (2013) [19] to retain beneficially the mineral and vitamin contents in nettle leaves. Furthermore, utilizing a pretreatment such as blanching was found to improve the product quality by inhibiting enzymatic activity as well as the drying efficiency, facilitating mass transfer [20,21]. However, no studies were found to systematically characterize the effects of pretreatment in combination with drying conditions on the techno-functional properties in relation to processing factors.

The present work aims to evaluate the feasibility of using blanching pretreatment in combination with two types of drying to obtain nettle powder as a potential additive or functional ingredient. The paper wants to contribute to the existing studies by enriching the available knowledge with information on some physicochemical, techno-functional, and nutritional properties of the obtained nettle powders.

## 2. Materials and Methods

### 2.1. Nettle Powder Preparation

Wild stinging nettle leaves were collected from Latium region (42.7477° N, 11.8630° E), Italy, in multiple harvests (3 lots) during the period of June–July 2019. Each set of harvested leaves was manually separated from the stem, cleaned, and split into two batches; one was not blanched (unblanched, U), while the other was blanched (B). The leaves were blanched using a sous-vide cooker (Model 10030542, Klarstein, Berlin, Germany) at 90 °C for 1 min, cooled in ice-cold water for 1 min, and drained. The optimal combination of blanching temperature and time was chosen from preliminary tests in agreement with Moscetti et al. (2017) [22]. Each batch of leaves were minced using a domestic blender and split in two equal parts on weight basis. One part was convective dried (CD) at 40 °C for about 12–14 h in a cabinet drier (Innotech, Leonberg, Germany), with an air flow of 1 m s^−1^. The other part was frozen at −80 °C and freeze-dried (Modulyo, Edwards) at −48 to −55 °C for 28 h.

The dried leaves after each treatment were ground in a laboratory scale mill (MF 10, IKA^®^-Werke, Staufen, Germany) using a 0.50 mm sieve with minimal heat production. The obtained nettle powders, namely UCD (unblanched, convective-dried), UFD (unblanched, freeze-dried), BCD (blanched, convective-dried), and BFD (blanched, freeze-dried) were weighed and stored in airtight glass bottles in dark at ≈4 °C until further analyses.

The nettle powder yield was calculated as the amount of powder obtained after milling to that of the non-blanched or blanched material prior to drying and expressed as g NP/100 g of material used.

### 2.2. Physicochemical and Techno-Functional Characteristics

The moisture content of both fresh leaves and differently dried powders were determined by the hot-air oven method (AOAC, 2000) [23] at 105 °C. Water activity (a_w_) was measured using a benchtop water activity meter (Acqualab, Decagon devices Inc., Pullman, WA, USA). The bulk density (*ρ*_β_, g cm^−3^) was read as the loose volume of 5 g of NP weighed into a 50 mL graduated cylinder. The cylinder was tapped repeatedly from a standard height as described by Caparino et al. (2012) [24], and the leveled volume was used to calculate the tapped bulk density (*ρ*_T_, g cm^−3^). The Carr Index (CI, Equation (1)) and Hausner Ratio (HR, Equation (2)) to evaluate the flowability and cohesiveness were calculated according to the equations given by Koç and Dirim (2018) [25]:

(1) $$Carr\;Index\;\left(CI\right)=\frac{\left(\rho_{T}-\rho_{B}\right)}{\rho_{T}}\;\times \;100,$$

(2) $$Hausner\;Ratio\;\left(HR\right)=\frac{\rho_{T}}{\rho_{B}},$$

Hygroscopicity was determined according to Koç and Dirim (2018) [25] with pre-weighed samples (0.5 g each) placed over a saturated solution of NaCl (R.H. 75%) at 20 ± 1 °C, weighed after 8 days. Results were expressed as moisture adsorbed in g per 100 g of NP.

Water-holding capacity (WHC) and water solubility index (WSI) were determined according to Ahmed et al. (2014) [26] on 0.5 g samples added with 10 mL of deionized water with few modifications. The samples were agitated mechanically (CertoMat, Germany) for 6 h at 20 °C, followed by centrifugation (NEYA 16R, Remi Elektrotechnik Ltd., Vasai, India) at 8000× *g*, 15 °C for 15 min. The residue in centrifuge tubes (50 mL) was weighed, and the WHC was calculated as weight of water held per gram of sample (g water g^−1^ NP, dw). The supernatant collected was dried at 105 °C until constant weight, and the dried residue weight was used to calculate the WSI (%).

The color of fresh leaves and powders was measured in the CIELab color space using a colorimeter (CM-2600d, Konica Minolta, Japan) with a D65 illuminant. The chromatic parameters were expressed as lightness (L*), redness (a*), yellowness (b*), chroma (C*), and hue angle (h) as well as CIELab color difference (ΔE*) of dried powders against fresh leaves [22].

### 2.3. Phytochemicals

Chlorophyll a (Chl a), chlorophyll b (Chl b), and total carotenoids (TC) were determined by procedure adapted from Wellburn (1994) [27] with some modifications: briefly, 100 mg of sample was weighed, added with 10 mL of acetone (99% pure), and extracted with ultrasound for 2 min (1st extraction). The extraction was repeated, increasing the time to 5 min for 2nd and 3rd extractions and 10 min for 4th and 5th extractions. The supernatant was pooled, made up to 50 mL, and centrifuged at 1000× *g* for 5 min at 15 °C. Subsequently, aliquots were read for absorbance in range of 380 to 750 nm. Pigment concentrations (µg mL^−1^ extract) and contents (mg 100 g^−1^ of NF, dw) were calculated according to Lichtenthaler and Buschmann (2001) [28] using the equation for acetone and Đurović et al. (2017) [8] using Equations (3)–(6),

(3) $$Chlorophyll\;a\;\left(\frac{\mu g}{mL\;of\;extract}\right)=11.24{\;A}_{661.6}-2.04{\;A}_{644.8},$$

(4) $$Chlorophyll\;b\;\left(\frac{\mu g}{mL\;\;of\;extract}\right)=20.13\;A_{644.8}-4.19A_{661.6},$$

(5) $$Total\;Carotenoids\left(\frac{\mu g}{mL\;\;of\;extract}\right)=\left(1000\;A_{470}-1.9\;c_{a}-63.14c_{b}\right)/214,$$

(6) $$m\;\left(\frac{mg\;of\;pigment}{g\;of\;NP}\right)=\frac{C\times V\times D_{f}}{G1000},$$

In Equation (6), *C* = concentration of chlorophyll a (*c_a_*), b (*c_b_*), or total carotenoids calculated using formulas (3)–(5); *V* = volume of acetone; *D_f_* = dilution factor; *G* = initial mass of the NP sample. Final values were presented as mg pigment/100 g nettle powder.

### 2.4. Antioxidant Capacity

One hundred milligrams of samples were added with 10 mL of methanol:water (95:5 *v*/*v*) solution in an orbital shaker (mod. 711, Tecnochimica Moderna S.r. l, Italy) in dark at ambient temperature for 24 h. The extract was centrifuged (mod. PK121R, ALC, Italy) at 8000× *g* for 5 min at 15 °C. The supernatant was collected and stored at −80 °C until further analyses. In the case of TPC, only one extraction was used: preliminary tests demonstrated that additional steps did not significantly improve phenols extractability.

Total phenolic content (TPC) was determined by the Folin–Ciocâlteu standard method with modifications [29]. The assay was conducted by mixing 4 mL of deionized water, 0.25 mL of diluted extract, 0.25 mL of diluted Folin–Ciocâlteu reagent (1:1), and 0.5 mL of 30% (*w*/*v*) Na_2_CO_3_. The mixture was stored in dark at room temperature for 30 min, and an aliquot was measured for absorbance at 725 nm using UV-spectrophotometer (UVikon-942, Kontron Instruments, Ztirich, Switzerland). The results were expressed as mg of gallic acid equivalents (GAE) per gram of sample, dw.

Antioxidant capacity was assessed by both ferric reducing antioxidant power (FRAP) and Trolox equivalent antioxidant capacity (TEAC) assays.

The FRAP assay was performed according to the method described by Benzie and Strain (1999) [30] adapted for 96-well plates and automatic reader (Infinite 2000, Tecan, Salzburg, Austria): 160 µL of FRAP assay solution (consisting of 20-mM ferric chloride solution, 10-mM TPTZ solution, and 0.3-M acetate buffer, pH 3.6) was freshly prepared and mixed with 10 µL of diluted sample (varied dilutions among samples, ratios not shown), standard or blank and dispensed into each well of a 96-well plate. The absorbance was measured at 595 nm after an incubation of 30 min in dark at 37 °C.

The TEAC assay was carried out using the OxiSelectTM TEAC Assay Kit (Cell Biolabs Inc., San Diego, CA, USA) according to the manufacturer instructions: 150 µL of ABTS reagent diluted 1:50 times in 75% ethanol freshly prepared was added to 25 µL of diluted sample (varied dilutions among samples, ratios not shown) and pipetted into each well of a 96-well plate. After 5 min incubation on an orbital shaker, the absorbance was measured at 405 nm. Results were expressed as mM of Trolox equivalents (TE) per gram of sample, dw.

### 2.5. Mineral Content

Monovalent and divalent cations (i.e., Na^+^, K^+^, Mg^2+^ and Ca^2+^) were determined by Ionic Chromatography equipped with a conductivity detector (IC-CD) according to the method described by Cataldi et al. (2003) [31]. Briefly, 4.0 mL of 5.0-mM HCl was added to ≈15 mg of sample, agitated on a shaker for 15 min, and centrifuged at 3000 rpm for 10 min. The resulting supernatant was diluted, filtered, and injected in the ionic chromatography system consisting of a LC-10ADvp solvent delivery pump and a CDD-10Avp conductivity detector (Shimadzu Corporation, Japan). The cation separation was carried out with a Universal Cation HR column (4.6 mm × 100 mm; Alltech Associates Inc., Deerfield, IL, USA) and eluted with aqueous 1.5-mM H_2_SO_4_ at a flow rate of 1 mL min^−1^.

Total iron quantification was performed using an inductively coupled plasma optical emission spectrometer (ICP-OES, Optima 8000 DV, PerkinElmer, Waltham, MA, USA) with an axially viewed configuration, equipped with an ultrasonic nebulizer, quartz torch, and quartz detector operating in the following conditions: RF-power of 1450 W, auxiliary gas flow rate of 0.3 L min^−1^, plasma gas flow rate of 10 L min^−1^, nebulizer flow rate of 0.65 L min^−1^, and sample aspiration flow rate of 1.5 mL min. The external calibration solutions were prepared from standard certified elemental solutions (CaPurAn) and Milli-Q water containing 3% HNO_3_ to get a range of concentrations (0.5 to 40 mg L^−1^). The sample (200 mg) was subjected to microwave-assisted (Mars plus CEM, Bergamo, Italy) acid digestion by the addition of 7.5 mL HNO_3_, 0.5 mL HCl, and 2.0 mL H_2_O_2_ (30%). A one-step heating program was used for 37 min from 25 to 180 °C and 15 min at 180 °C at 1200 W. After cooling, the digested sample solutions were carefully transferred into 25 mL volumetric flasks to quantify prior to analysis by ICP-OES. Reagent blanks were prepared containing the same reagents as the samples.

### 2.6. Statistical Analysis

All measurements were carried out in triplicates except for density, which was duplicated. The data were elaborated by two-way analysis of variance (ANOVA) with Tukey’s honest significant differences (HSD) as post hoc test (α = 0.05) using R-software (R-studio, version 3.6.2) and the ‘agricolae’ R-package. Results were arranged in tables and expressed as mean ± standard deviation for both main effects (i.e., pretreatment and drying) and the interaction effect (i.e., pretreatment × drying).

## 3. Results and Discussion

### 3.1. Physicochemical and Techno-Functional Characteristics

#### 3.1.1. Moisture Content and Water Activity

The nettle powders obtained (Section 2.1) were visually uniform without aggregates and ranged from a dark green to an olive-green color. The yields ranged from 11.29 to 14.81 g 100 g^−1^ and were lowered by blanching pretreatment as expected due to the leaching of solids and lower moisture content post-processing (Table 1). The moisture content lowered from 87 to 89 g 100 g^−1^ in fresh nettle leaves to 3 to 11 g 100 g^−1^ in the dried powders and was apparently affected by the interaction of pretreatment and drying conditions (*p* < 0.05, Table 1). The use of freeze drying, particularly in combination with blanching, lowered the moisture content as opposed to that observed by Shonte et al. (2020) [5], wherein freeze-dried (6.4 g 100 g^−1^) nettles registered higher values than the oven-dried (3.4 g 100 g^−1^) nettles. This difference observed might be attributed to the size reduction of the nettle leaves prior to drying and the drying conditions used in this study. The water activity was in accordance with the moisture content with values below 0.60 indicative of microbial and bio-chemical stability.

#### 3.1.2. Flow Properties

Table 1 reports the flow properties affecting the product reconstitution, packaging, handling, and storage properties of the NPs. The bulk density (*ρ*_β_) was significantly influenced by the drying method irrespective of blanching factor, whereas the tapped bulk density (*ρ*_T_) by the interaction of factors with FD nettle powders having lower values than the CD nettle powders (*p* < 0.05).

The differences in *ρ*_β_ can be attributed to the drying method, wherein the sublimation of ice at temperatures lower than initial product’s glass transition temperature in freeze-drying resulted in a porous structure. This was primarily due to the absence of liquid transfer from the layers to the surface of the plant tissues as in CD, thereby preventing the tissue shrinkage and structural collapse. Similar observations were made in FD black plum powders [32] and freeze-dried mango powders [24] with *ρ*_β_ values of 387 and ≈400 kg m^−3^, respectively, which were higher than those of freeze-dried NPs (≈200 kg m^−3^). As for the tapped bulk density (*ρ*_T_), the values were in general higher than *ρ*_β_ due to compact arrangement of particles as also observed in dried maple syrup by Bhatta et al. (2019) [33]. The values were double those of their respective *ρ*_β_ values in case of FD powders indicative of voids and a higher amount of occluded air that pack loosely in comparison to CD powders (Table 1).

Furthermore, the handling properties namely, flowability and cohesiveness given by Carr Index (CI) and Hausner Ratio (HR) behaved in a similar fashion to that of *ρ*_T_ and *ρ*_β_ values, respectively (Table 2). Freeze-dried NPs, irrespective of the pretreatment, exhibited high cohesiveness (HR > 1.4, poor) and high compressibility (CI > 45%, poor) despite low moisture contents, which can be attributed to their lower density and increased inter-particle forces as compared to that of CD powders [33]. This indicates that that pure FD nettle powder can be less efficient in production and processing than CD nettle powder, potentially leading to additional load on the sieves, downtime due to clogged conveyor lines, improper discharge from bins, and process downtime [34]. A good understanding of the flowability of an ingredient is fundamental to adapt processing conditions and, thus, obtain a high-quality product [34]. In addition, Bhatta et al. (2019) [33] observed better flow characteristics for spray-dried maple syrup powders with higher moisture, wherein the moisture was hypothesized to act as a lubricant, thereby improving the flowability.

#### 3.1.3. Hygroscopicity, Water-Holding Capacity and Water Solubility Index

Hygroscopicity, as a critical parameter affecting the flowability and storage stability, ranged from 4.8 to 12.5 g 100 g^−1^ and was significantly influenced by the drying method (Table 2). The observed values were only slightly higher than the cut-off values (5.13 to 9.38 g 100 g^−1^) arbitrarily used by Caparino et al. (2012) [24] for mango powders (16 to 20 g 100 g^−1^). Freeze-dried NPs presented higher values consistent with their lower water activity and higher moisture gradient in test environment resulting in higher moisture adsorption. This hygroscopic behavior of FD nettle powders further confirms their poor flowability and higher cohesiveness.

WHC and WSI, affecting the rehydration capacity and formulation characteristics (e.g., the amount of water added to a formulation), were influenced by the interaction of factors with blanching and freeze drying synergistically increasing the values (Table 2). This can be attributed to the fact that although blanching degrades the cell structure, freeze drying induces a higher porosity of the cells with respect to that of convective drying. Ahmed et al. (2020) [35] observed similar trend in banana powders with FD powders (3.2 g g^−1^, db) having notably higher values due to their finer particle size in comparison to the tray-dried samples (2.73 g g^−1^, db).

As for the Water Solubility Index, CD powder had a lower value than FD powder only for unblanched samples. It can be explained as a result of interaction among nutrients due to exposure to a higher temperature, as seen in Correa et al. (2011) [36]. No differences between drying methods were noted for blanched samples, although blanching significantly reduced WSI as already observed by Fombang et al. (2017) [37] in Moringa leaves.

#### 3.1.4. Colorimetric Parameters

The colorimetric parameters of fresh nettle leaves used both as reference and to calculate total color difference (ΔE*) were L_0_* = 37.10 ± 2.71, a_0_* = −9.65 ± 0.98 and b_0_* = −19.41 ± 2.12. The chromatic parameters of the NPs were different from those of fresh leaves and significantly affected by the interaction of factors (Table 3). The ΔE* value was lowest for BFD powder, whereas UFD powder showed highest values indicating that blanching limited the color changes during drying.

In general, the L* values were higher in NPs than fresh leaves owing to water loss as also observed in dried curcuma leaves [38]. Significantly higher L* value was observed only for UFD powder, which probably influenced the ΔE* value. This might be due to the scattering of the incident light from the increased particle surfaces throughout a range of angles [39] relative to the smaller size of the powder particles [40].

As for the redness (a*) parameter, the lowest value was observed in BFD powders followed by BCD, UFD, and UCD. This indicates that blanching, particularly in combination with FD, reduced the a* value by limiting the enzymatic changes and thermal degradation of the pigments [41]. With respect to yellowness, FD powders showed higher b* values indicating a higher retention of carotenoids due to reduced heat exposure.

Furthermore, higher hue angle values of blanched and freeze-dried NPs confirm their higher green tonality relative to lower a* values. As for the color intensity, the chroma had a similar trend as that of b* values. Relatively lower color saturation was observed in UFD with higher L* values in comparison to BFD powders, confirming their lower greenness.

### 3.2. Bio-Active and Nutritional Characteristics of Differently Processed NPs

#### 3.2.1. Phytochemicals

Nettle leaves were found to be rich sources of commercially valued chlorophylls with Chl a and Chl b ratios ranging from 2.51 to 4.48 in fresh and blanched leaves [42]. In the present study, the Chl a to b ratio ranged from 2.3 to 2.6, wherein the Chl a content was affected by both the factors (blanching and drying), and Chl b content was affected by only that of the drying method (Table 4). In case of Chl a, blanched NPs were observed to have higher values that can be attributed to the protective effect of blanching that was found to limit enzyme activity and pheophytin formation during drying in mint and basil leaves [43,44].

As for drying method, generally, both chlorophyll a and b were positively influenced by freeze-drying method with higher values than convection-dried powders. This can be attributed to the higher pigment retention in FD powders due to the low temperature drying, resulting in limited damage and better extractability of the chlorophylls as consequence of a more porous product, meaning that solvents can easily penetrate the matrix and extract more phytochemicals [16,44]. Moreover, the low temperatures in freeze drying prevented the degradation of chlorophylls to pheophytins due to heat exposure. Among the individual chlorophylls, Chl a registered a significant loss in convective-dried NPs due to its thermolabile nature. A similar loss of chlorophylls was observed in oven-dried and freeze-dried nettle and kale leaves by Branisa et al. (2017) [16] and by Korus et al. (2013) [44], respectively.

The total carotenoids content in previous studies ranged between 5.14 and 262 mg 100 g^−1^ in nettle leaves depending on the processing methods, maturity, and other agronomic factors [8,9,16]. In this study, the TC ranged from 101.39 to 200.63 mg 100 g^−1^ NP, dw (Table 4) with blanched powders retaining higher contents, particularly when freeze-dried. The effect of blanching can be attributed to the increased extractability due to cell disruption as observed in broccoli by-products [45] and/or to the inactivation of oxidative enzymes potentially involved in pigments degradation during drying. Moreover, the low temperatures and sub-atmospheric pressures in freeze drying better retained carotenoids, which are sensitive to heat, light, and oxygen, as also observed by Branisa et al. (2017) [16] and Shonte et al. (2020) [5].

Overall, the studied pigments were better preserved in the order BFD > UFD > BCD > UCD and are in accordance with the trend of chromatic parameters (a* and b*), confirming the positive effect of blanching and freeze-drying processes on color and pigment stability.

#### 3.2.2. Antioxidant Activity

**Figure 1:**
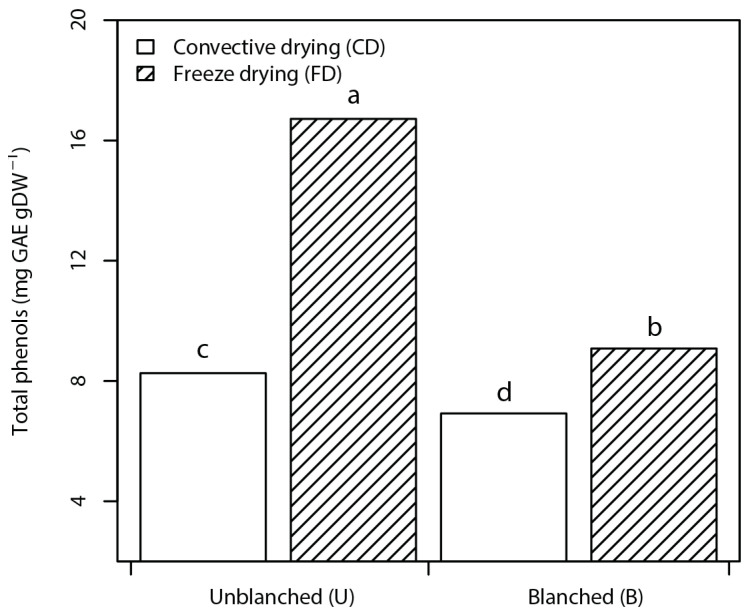
Bar plots of the interaction effect of pretreatment and drying factors on total phenolic content (TPC) of nettle powders. Data are mean ± standard deviation of the mean. Mean values belonging to the same factor without common letters are statistically different according to HSD (*p* ≤ 0.05).

Nettles are good sources of phenolic compounds with contents ranging from 29 to 129 mg GAE g^−1^ of nettle (dw) and exhibit various biological activities attributed to hydro-cinnamic acids, flavonoids, and tannins [5,7,8,18]. In this study, the phenol contents ranged from 6.92 to 16.72 mg GAE g^−1^ of NP (dw) and were significantly affected by interaction of factors. UFD had the highest total phenols followed by BFD–UCD, with the least values in BCD powders (Figure 1).

Similar observations were made by Korus (2011) [20] in kale leaves with the highest values of phenolic content for unblanched, freeze-dried leaves and the lowest values for blanched, air-dried kale. The observed trend can be attributed to the combined effect of blanching and CD lowering the phenols due to component leakage and thermal degradation, respectively. However, higher phenol contents in nettles oven-dried at 70 °C observed by Shonte et al. (2020) [5] were attributed to the condensation of tannins resulting in higher values than freeze-dried nettles.

**Figure 2:**
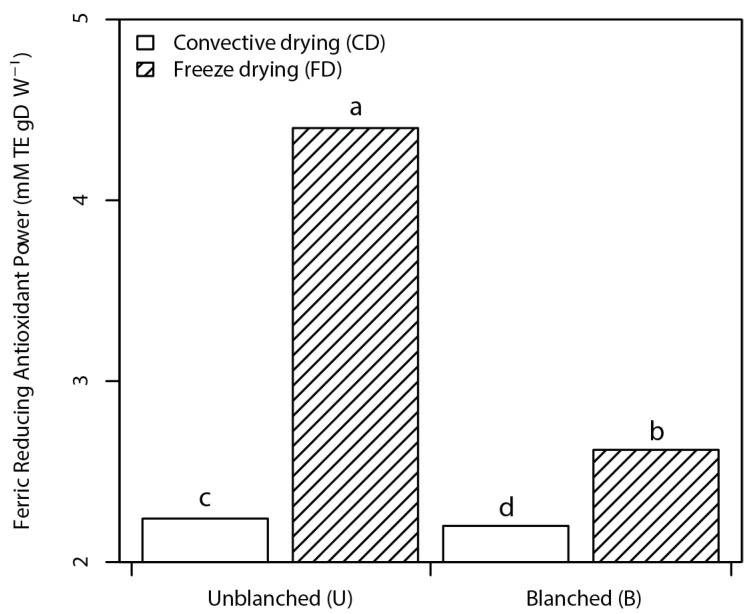
Bar plots of the interaction effect of pretreatment and drying factors on ferric reducing antioxidant power (FRAP) of nettle powders. Data are mean ± standard deviation of the mean. Mean values belonging to the same factor without common letters are statistically different according to HSD (*p* ≤ 0.05).

**Figure 3:**
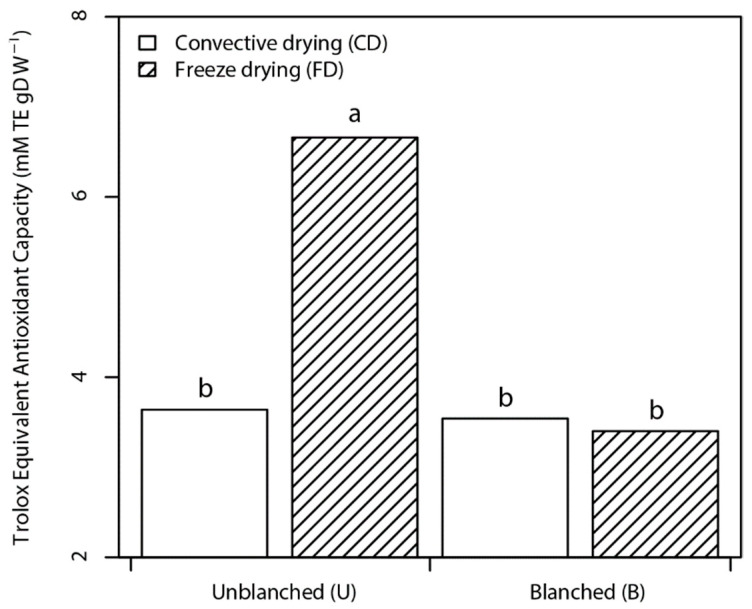
Bar plots of the interaction effect of pretreatment and drying factors on the Trolox equivalent antioxidant capacity (TEAC) of nettle powders. Data are mean ± standard deviation of the mean. Mean values belonging to the same factor without common letters are statistically different according to HSD (*p* ≤ 0.05).

The total antioxidant capacity as reducing capacity and scavenging ability as presented in Figure 2 and Figure 3 varied between 2.20 to 4.40 and 3.40 to 6.67 mM TE g^−1^ NP (dw) for FRAP and TEAC, respectively.

Subsequently, higher total antioxidant capacity was observed in UFD nettle powder owing to its higher phenolic content. In addition, blanching coupled with CD resulted in lower FRAP values in correspondence to the TPC values. However, no significant differences were found between TEAC values of BCD, BFD, and UCD powders, which can be attributed to the fact that TEAC does not consider metal chelation as well as the different polyphenol structure and assay compound reactivity.

### 3.3. Mineral Content

Table 5 summarizes the contents of selected minerals calcium, iron, magnesium, potassium, and sodium as affected by the pretreatment and/or drying factors.

Calcium content was not affected by either factors and was lower than that reported by Đurović et al. (2017) [8] in shade-dried leaves (28.60 mg g^−1^, dw) and Shonte et al. (2020) [5] in oven/freeze-dried nettles (21–23 mg g^−1^, dw). As for magnesium and potassium, the blanched powders had lower values due to the higher susceptibility of Mg and K to leaching and were not subjected to the chelating effects of organic matrix unlike cations of group II. The higher quantity of Mg in FD powder might be attributed to the increased extractability. As for Na content, it relatively increased probably due to better extractability as also observed in nettles blanched or cooked in salt water by Rutto et al. (2013) [19].

Iron is the only trace element, and the mean values were 32.40 ± 5.61 mg 100 g^−1^ (dw) in unblanched and 5.06 ± 1.36 mg 100 g^−1^ (dw) in blanched NPs, irrespective of the drying method utilized with blanching adversely affecting the values [19].

In general, the mineral contents of NPs in this study are not in agreement with the data reported in literature [7,8] owing to the variations in plant maturity and other agronomic factors. However, considering the mineral contents it can be said that 1 g of nettle powder intake can provide up to 2.14% of calcium, 0.75% of magnesium, 0.01% of sodium, and 0.60% of potassium of the required intakes for an average adult above 25 years as suggested by EFSA [46].

## 4. Conclusions

The results of this study showed that nettle leaves can be effectively used to produce powders by blanching as well as drying processes. The blanching and drying methods used (CD and FD) influenced the physicochemical, technological, and functional quality of the powders. In particular, the use of blanching resulted in an improvement of the product physicochemical stability in terms of color and pigment retention. Regarding the drying process, although convective drying resulted in superior flow properties, freeze drying provided higher antioxidant potential, pigments, and color retention. Although an expensive and time-consuming process, freeze-dried powders are more suitable for processing to have higher availability of bioactive components and nutrients. Although the results obtained can be interesting for academic or industrial uses, further studies are needed to confirm these findings and deeply explore pros and cons in the usage of nettle powder as an ingredient/additive for novel foods (e.g., bakery products). Additional studies are being carried out (i) to characterize the phenolic profile of nettle powders through HPLC; (ii) to evaluate the effects of blanching and drying on the product stability during storage; and (iii) to evaluate the anti-diabetic effect of nettle powder after incorporation into a food matrix by analyzing the content of cyclic proteins and performing in vivo tests, which were beyond the scope of the presented study.

## Figures and Tables

**Table 1:** The interaction of pretreatment and drying factors on the physicochemical and technological properties of nettle powders. Data are mean ± standard deviation of the mean. Mean values belonging to the same factor without common letters are statistically different according to the Honestly Significant Difference or HSD (*p* ≤ 0.05).

Factor	Yield (%)	Moisture Content * (g 100 g^−1^)	Water Activity (a_w_)	Bulk Density (g cm^−3^)	Tapped Density (g cm^−3^)
**Pretreatment (PR)**					
Unblanched (U)	13.99 ± 1.93a	8.06 ± 2.28	0.42 ± 0.12	0.30 ± 0.13	0.44 ± 0.10
Blanched (B)	11.67 ± 1.14b	6.79 ± 3.13	0.34 ± 0.17	0.30 ± 0.14	0.45 ± 0.13
*p* value	≤0.05	≤0.05	≤0.05	ns	ns
HSD	0.035				
**Drying (DR)**					
Convective drying (CD)	13.05 ± 2.24	9.89 ± 0.29	0.51 ± 0.02	0.41 ± 0.02a	0.54 ± 0.02
Freeze drying (FD)	12.61 ± 1.77	4.96 ± 1.12	0.24 ± 0.07	0.18 ± 0.01b	0.35 ± 0.02
*p* value	ns	≤0.05	≤0.05	≤0.05	≤0.05
HSD				0.001	
**PR × DR**					
U × CD	14.81 ± 1.27	10.13 ± 0.14a	0.53 ± 0.01a	0.41 ± 0.02	0.53 ± 0.02b
U × FD	13.18 ± 2.38	5.98 ± 0.02c	0.30 ± 0.01c	0.19 ± 0.01	0.36 ± 0.01c
B × CD	11.29 ± 1.30	9.64 ± 0.10b	0.49 ± 0.01b	0.42 ± 0.01	0.56 ± 0.01a
B × FD	12.04 ± 1.07	3.93 ± 0.07d	0.18 ± 0.02d	0.18 ± 0.01	0.34 ± 0.01c
*p* value	ns	≤0.05	≤0.05	ns	≤0.05
HSD		0.001	0.001		0.02

ns = no significant difference; * nettle leaves initial moisture content 86.55 ± 0.84 (U) and 88.52 ± 1.03 (B) g 100 g^−1^.

**Table 2:** The interaction effect of pretreatment (PR) and drying (DR) factors on the physicochemical and technological properties of nettle powders. Data are mean ± standard deviation of the mean. Mean values belonging to the same factor without common letters are statistically different according to the Honestly Significant Difference or HSD (*p* ≤ 0.05).

Factor	Carr Index (%)	Hausner Ratio	Hygroscopicity (g H_2_O 100 g DW^−1^)	WHC (g H_2_O g DW^−1^)	WSI (%)
**Pretreatment (PR)**					
Unblanched (U)	36.00 ± 15.03	1.63 ± 0.38	7.49 ± 2.90	6.18 ± 0.32	11.90 ± 1.47
Blanched (B)	36.00 ± 12.73	1.60 ± 0.35	8.59 ± 4.27	6.14 ± 1.19	5.97 ± 0.57
*p* value	ns	ns	ns	ns	≤0.05
HSD					
**Drying (DR)**					
Convective drying (CD)	24.00 ± 1.15	1.30 ± 0.01b	4.85 ± 0.42b	5.49 ± 0.49	8.14 ± 2.73
Freeze drying (FD)	48.00 ± 1.63	1.93 ± 0.05a	11.23 ± 1.66a	6.83 ± 0.43	9.72 ± 3.82
*p*-value	≤0.05	≤0.05	≤0.05	≤0.05	≤0.05
HSD		0.001	0.001		
**PR × DR**					
U × CD	23.00 ± 0.01b	1.30 ± 0.01	4.86 ± 0.61	5.92 ± 0.12c	10.62 ± 0.58b
U × FD	49.00 ± 1.41a	1.95 ± 0.07	10.12 ± 0.14	6.45 ± 0.17b	13.17 ± 0.39a
B × CD	25.00 ± 0.01b	1.30 ± 0.01	4.84 ± 0.24	5.06 ± 0.20d	5.67 ± 0.06c
B × FD	47.00 ± 1.41a	1.90 ± 0.01	12.34 ± 1.78	7.22 ± 0.01a	6.27 ± 0.73c
*p* value	≤0.05	ns	ns	≤0.05	≤0.05
HSD	0.05			0.001	0.01

ns = no significant difference; WHC = water-holding capacity; WSI = water solubility index.

**Table 3:** The interaction effect of pretreatment (PR) and drying (DR) factors on colorimetric parameters of the nettle powders obtained from different treatments. Data are mean ± standard deviation of the mean. Mean values belonging to the same factor without common letters are statistically different according to HSD (*p* ≤ 0.05).

Factor	Luminance (L*)	Redness (a*)	Yellowness (b*)	Chroma (C*)	Hue Angle (h)	ΔE*
**Pretreatment (PR)**						
Unblanched (U)	43.59 ± 2.87	−3.76 ± 0.70	18.77 ± 1.12	19.15 ± 1.23	101.27 ± 1.42	9.14 ± 1.46
Blanched (B)	41.14 ± 0.90	−7.21 ± 1.82	18.50 ± 2.59	19.87 ± 3.07	111.01 ± 2.24	5.61 ± 0.88
*p* value	≤0.05	≤0.05	ns	≤0.05	≤0.05	≤0.05
HSD						
**Drying (DR)**						
Convective drying (CD)	40.89 ± 1.13	−4.34 ± 1.33	16.96 ± 0.91	17.56 ± 0.58	104.48 ± 4.92	7.14 ± 0.97
Freeze-drying (FD)	43.85 ± 2.46	−6.64 ± 2.45	20.32 ± 0.64	21.46 ± 1.34	107.80 ± 5.76	7.62 ± 3.05
*p* value	≤0.05	≤0.05	≤0.05	≤0.05	≤0.05	ns
HSD						
**PR × DR**						
U × CD	41.15 ± 1.48b	−3.13 ± 0.02a	17.76 ± 0.06c	18.03 ± 0.05c	99.99 ± 0.10d	7.93 ± 0.70b
U × FD	46.02 ± 0.75a	−4.40 ± 0.10b	19.78 ± 0.22b	20.27 ± 0.21b	102.55 ± 0.34c	10.36 ± 0.66a
B × CD	40.62 ± 0.90b	−5.55 ± 0.17c	16.16 ± 0.40d	17.09 ± 0.43d	108.97 ± 0.22b	6.35 ± 0.16c
B × FD	41.67 ± 0.61b	−8.87 ± 0.11d	20.85 ± 0.38a	22.66 ± 0.39a	113.05 ± 0.14a	4.87 ± 0.53d
*p* value	≤0.05	≤0.05	≤0.05	≤0.05	≤0.05	≤0.05
HSD	0.01	0.001	0.001	0.001	0.001	0.001

ns = no significant difference; ΔE* = CIELab color difference.

**Table 4:** The interaction effect of pretreatment (PR) and drying (DR) factors on chlorophylls and total carotenoid content of the nettle powders obtained from different treatments. Data are mean ± standard deviation of the mean. Mean values belonging to the same factor without common letters are statistically different according to HSD (*p* ≤ 0.05).

Factor	Chlorophyll a (mg 100 g DW^−1^ NP)	Chlorophyll b (mg 100 g DW^−1^ NP)	Total Carotenoids (mg 100 g DW^−1^ NP)
**Pretreatment (PR)**			
Unblanched (U)	512.68 ± 105.51b	215.35 ± 38.53	128.13 ± 29.58
Blanched (B)	585.31 ± 155.52a	229.08 ± 55.07	162.49 ± 42.31
*p* value	≤0.05	ns	≤0.05
HSD	0.005		
**Drying (DR)**			
Convective drying (CD)	432.61 ± 21.35b	180.73 ± 3.98b	112.88 ± 13.19
Freeze-drying (FD)	665.38 ± 74.29a	263.70 ± 22.02a	177.75 ± 25.99
*p* value	≤0.05	≤0.05	≤0.05
HSD	0.001	0.001	
**PR × DR**			
U × CD	417.54 ± 19.63	180.53 ± 5.67	101.39 ± 4.97d
U × FD	607.82 ± 17.09	250.17 ± 6.50	154.87 ± 4.30b
B × CD	447.68 ± 8.53	180.94 ± 2.72	124.36 ± 3.77c
B × FD	722.94 ± 59.70	277.23 ± 24.92	200.63 ± 10.00a
*p* value	ns	ns	≤0.05
HSD			0.014

ns = no significant difference.

**Table 5:** The interaction effect of pretreatment (PR) and drying (DR) factors on mineral content of nettle powders obtained by different processing methods. Data are mean ± standard deviation of the mean. Mean values belonging to the same factor without common letters are statistically different according to HSD (*p* ≤ 0.05).

Factor	Ca (mg g DW^−1^ NP)	K (mg g DW^−1^ NP)	Mg (mg g DW^−1^ NP)	Na (mg g DW^−1^ NP)
**Pretreatment (PR)**				
Unblanched (U)	16.83 ± 0.97	23.1 ± 1.26a	2.59 ± 0.35	0.28 ± 0.02b
Blanched (B)	15.99 ± 1.37	10.85 ± 0.88b	2.26 ± 0.57	0.38 ± 0.03a
*p* value	ns	<0.05	<0.05	<0.05
HSD		0.001	0.032	0.001
**Drying (DR)**				
Convective drying (DR)	16.00 ± 0.78	17.17 ± 6.45	2.04 ± 0.30b	0.33 ± 0.07
Freeze drying (FD)	16.82 ± 1.50	16.78 ± 7.11	2.81 ± 0.27	0.33 ± 0.04
*p* value	ns	ns	<0.05	ns
HSD			0.001	
**PR × DR**				
U × CD	16.25 ± 0.94	22.98 ± 1.59	2.29 ± 0.16	0.26 ± 0.02
U × FD	17.41 ± 0.67	23.22 ± 1.19	2.89 ± 0.07	0.29 ± 0.01
B × CD	15.75 ± 0.67	11.36 ± 0.56	1.79 ± 0.12	0.39 ± 0.03
B × FD	16.23 ± 2.02	10.34 ± 0.91	2.73 ± 0.39	0.36 ± 0.02
*p* value	ns	ns	ns	ns
HSD				

ns = no significant difference.
